# Untangle those stethoscopes; never too early to start reflecting! Qualitative review of a reflective practice group for clinical undergraduate medical students

**DOI:** 10.1192/j.eurpsy.2021.1775

**Published:** 2021-08-13

**Authors:** E. Jordan, S. Patel, E. Mcguire, P. Noonan, G. Mccarthy

**Affiliations:** 1 Sligo Medical Academy, NUIG, Sligo, Ireland; 2 Aamhu - Psychiatry, University Hospital Galway, Galway, Ireland; 3 Gp, The Medical Centre, CO. LEITRIM, Ireland

**Keywords:** Medical Education, COVID-19, Reflective Practice

## Abstract

**Introduction:**

Equipping our medical students with as many tools as possible to cope with the challenges that they will inevitably face has never been more important than it is today.

**Objectives:**

The aim of this study was to examine the effectiveness of a reflective practice (RP) group for medical students, particularly with adaptation to COVID-19 and transition to video.

**Methods:**

A pilot programme of RP for 3^rd^ year medical students commencing their clinical placement was run by the Sligo Medical Academy, NUIG in Ireland between January – April 2020. This group for nine students was initially run face-to-face but pivoted to an online group in March 2020 with the COVID-19 pandemic. Data was collected through one-to-one interviews with all student participants and the facilitator (n=10). Interviews were recorded and transcribed. Data were analysed using thematic content analysis.

**Results:**

Our analysis identified four main discussion themes: transition to clinical environment, gender in the workplace, building professional identity and family and support systems. The students who continued the RP group over zoom during the COVID-19 pandemic particularly identified with the theme of support systems and solidarity. The smooth transition to zoom and its effectiveness in a time of social distancing were discussed. Identified challenges related primarily to timing of the RP group, particularly after a full day of placements or time differences for international students overseas.

**Conclusions:**

Reflective practice programmes are not routinely offered to medical students in Ireland currently and this study gives recommendations on implementing and improving experiences of undergraduate training based on RP.

**Disclosure:**

No significant relationships.

